# Can a Byte Improve Our Bite? An Analysis of Digital Twins in the Food Industry

**DOI:** 10.3390/s22010115

**Published:** 2021-12-24

**Authors:** Elia Henrichs, Tanja Noack, Ana María Pinzon Piedrahita, María Alejandra Salem, Johnathan Stolz, Christian Krupitzer

**Affiliations:** Department of Food Informatics and Computational Science Lab, University of Hohenheim, 70599 Stuttgart, Germany; tanja.noack@uni-hohenheim.de (T.N.); ana.pinzon@uni-hohenheim.de (A.M.P.P.); maria.salem@uni-hohenheim.de (M.A.S.); johnathan.stolz@uni-hohenheim.de (J.S.)

**Keywords:** digital twins, food industry, food supply chain, cyber–physical systems, sensors, Internet-of-Things, survey

## Abstract

The food industry faces many challenges, including the need to feed a growing population, food loss and waste, and inefficient production systems. To cope with those challenges, digital twins that create a digital representation of physical entities by integrating real-time and real-world data seem to be a promising approach. This paper aims to provide an overview of digital twin applications in the food industry and analyze their challenges and potentials. Therefore, a literature review is executed to examine digital twin applications in the food supply chain. The applications found are classified according to a taxonomy and key elements to implement digital twins are identified. Further, the challenges and potentials of digital twin applications in the food industry are discussed. The survey revealed that the application of digital twins mainly targets the production (agriculture) or the food processing stage. Nearly all applications are used for monitoring and many for prediction. However, only a small amount focuses on the integration in systems for autonomous control or providing recommendations to humans. The main challenges of implementing digital twins are combining multidisciplinary knowledge and providing enough data. Nevertheless, digital twins provide huge potentials, e.g., in determining food quality, traceability, or designing personalized foods.

## 1. Introduction

With the evolution and digitalization towards Industry 4.0, the concept of creating digital copies of physical counterparts received entry to the industry [1]. In particular, the food industry is of special interest because it requires a high efficient use of the available resources [2]. Over time, food production systems have evolved alongside technological innovations, allowing for increased production, greater product variety, more resilient food stocks, and increased international trade. Yet, despite these advances, food systems around the world continue to face unprecedented challenges. Challenges such as climate change, pressure to feed a growing global population, and persistent global food waste pose significant threats to current food systems. In addition, there are growing societal demands for greater food provenance, traceability, and sustainability within the food system [3].

A key element of Industry 4.0 is the digital twin: a virtual model of a product or process created with data collected by sensors that enables simulations or real-time analyses of the status of production [1,4]. The use of digital twins seems beneficial in food processing for various reasons. The COVID-19 pandemic demonstrated the vulnerability of food supply resilience [5]. To ensure the supply of foods, production processes must allow high flexibility and adaptivity [6]. Furthermore, product quality is influenced by different quality levels of input materials. Especially in the case of seasonal fluctuations impacting raw material quality, an adjustment of parameters in the production process is essential. Introduction processes of new products could be simplified by a digital twin of already existing products. The digital twin is able to learn the correct process parameters for production and is used as the knowledge base within a self-adaptive software system [7]. However, a digital twin of food production has additional specific requirements compared to digital twins of the production of material goods [8]. Due to the variability of raw materials, these cannot be based only on the processing steps but must also take into account the chemical, physical, or (micro)biological properties of the food. Further, the technology can be applied to create a detailed digital model of the supply chain that integrates real-time and real-world data to respond to unexpected events and uncertainty within the supply chain.

This work aims to provide an overview of digital twin applications in the food industry and analyze their challenges and potentials. Therefore, we first present a taxonomy to differentiate underlying technologies and better understand the intended use of each digital twin. Second, a survey is executed to examine digital twin applications in the food supply chain (FSC). We target the FSC as it provides a link between all the key activities and processes involved in bringing a specific food product to market [9]. To meet these unprecedented challenges, FSCs and corresponding actors are turning to modern technology for assistance [10]. We classify the found applications of digital twins according to our taxonomy. Third, we investigate the key elements to implement digital twins in the FSC. Fourth, since the concept of digital twins is still young, we discuss the potentials of applying them in the food sector. Finally, we discuss the challenges of applying digital twins in the food industry. In summary, this paper contributes to the body of research by providing the following scopes:

- Classification of digital twins in the food sector.
- Overview of the application of digital twins in the food industry.
- Definition of the key elements for implementing a digital twin.
- Analysis of the potential of digital twins in the food industry.
- Description of challenges of applying digital twins in the food industry.

The remainder of the paper is structured as follows. Next, Section 2 explains several fundamentals related to the FSC, the digitalization of the food industry, and provides a definition of digital twins. Then, Section 3 presents the methodological approach for the literature review. Subsequently, Section 4 evaluates the literature review results and summarizes the key elements for implementing digital twins. We discuss the potentials and challenges of digital twins and their implementation in the food supply chain in Section 5. In Section 6, we discriminate this work against other publications in the field. Finally, Section 7 concludes this paper with a summary of our results.

## 2. Background

In the following Section 2.1, we first describe the underlying concept of the FSC, which we use to differentiate the stages in the food industry and to classify the digital twin applications in Section 4. Further, the role of digitalization in the food industry as well as related concepts are described in Section 2.2. Finally, Section 2.3 provides a definition of digital twins.

### 2.1. The Food Supply Chain

A supply chain (SC) is a network of actors structured around activities and processes that aim to satisfy given consumer demand by bringing products or services to market [11]. This network includes feedback and circular economy aspects, e.g., for sustainability reasons as the recycling of materials [12]. The actors within the SC are linked through upstream or downstream processes and activities that produce value in the form of finished products or services [11]. In the same sense, a FSC encompasses all activities involved in the creation and transformation of raw materials into food products as well as their retail and consumption [10]. FSCs do differ significantly from other SCs due to the complexity of producing, transporting, and managing food products [13].

**Figure 1:**
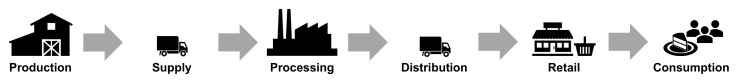
A simplified structure of the food supply chain (based on [10]) including the actors used to classify the digital twin applications within the scope of this work. The structure does not show any circular flows or side chains of by-, side-, or co-products, which would result in a value network rather than a straight-forwarded chain.

While it is important to consider not only the primary flow but also the tangential and secondary flows that are contained within the FSC, as these are opportunities to reduce food loss and waste through reuse and recycling [12], we focus on a simplified, linear, and straight forwarded structure of the FSC. This is sufficient for this survey since the focus is on single activities of the FSC that are present identically in the simplified FSC as well as in a circular view. Figure 1 provides an overview of the FSC and the main actors, to which the digital twin applications will be assigned. Commonly, the FSC begins with *production*, which is usually an agricultural farm, continues with *supply*, *processing*, *distribution*, and *retail* and ends with the *consumption*.

Worth mentioning is that the stages could be thereby divided into several processing or transportation sub-entities: For instance, Shoji et al. [14] investigate the cold chain of fruits and vegetables from a (farm to) packhouse to distribution to the retailer. The authors divide the transportation steps between the supply from packhouse to distribution center and from distribution center to the retailer. In our understanding, the packhouse would be part of the processing stage and both, the transportation and the distribution would be summarized as distribution.

It is crucial for SCs to be designed with consideration for uncertainty and risk, as mitigation measures and solutions must be developed to prevent disruptions to the SC [9]. Those disruptions impact the SC’s regular flow and affect the other actors directly [15]. In particular, the most frequent FSC disruptions are human error, communication misunderstandings, organizational process errors, and quality problems with goods received [16]. Consequently, disruptions may result in negative effects to the final product [9] regarding sustainability, safety, and quality [13].

Additionally, several challenges in the FSC occur during different stages [17]: the production estimation and optimization in the production stage, including the crop management and security and the livestock control; the production planning in the processing stage, regarding the post-harvest loss as well as demand prediction; the distribution, concerning route planning, prediction of SC risks and disruptions, and shelf-life prediction; and the consumption, representing consumer behavior, their dietary behavior, food loss and waste, or the prediction of the daily demand.

### 2.2. Industry 4.0 and Related Concepts

“Industry 4.0” is associated with the fourth industrial revolution. It combines technologies such as cyber–physical systems (CPSs), Internet of Things (IoT), and cloud computing. While the term Industry 4.0 is primarily used in Europe, the similar concept “Industrial Internet of Things” (IIoT) mainly used in the US describes advances in big data, cloud computing, and networking of machinery and systems in the industrial sector [18]. Based on CPSs and IoT, in Industry 4.0 manufacturing processes including logistics (i.e., SC management), services, and maintenance are efficiently synchronized [19]. Hence, Industry 4.0 does not focus on a single process or technology but integrates all processes resulting in a highly flexible and integrated optimized manufacturing process. The complete implementation of Industry 4.0 or IIoT would result in the smart factory: an integrated production process that is entirely self-organizing by the connected machines and intelligent software without any human interaction [20].

Further, modern FSCs make increasingly use of integrated information and communication technology (ICT) systems to assist with mitigating against uncertainty and risk, process optimization, and numerous other applications [11]. In addition, ICT systems are of particular interest for traceability and decision-making functions within the FSC [21]. Traceability is important to identify quality and safety concerns and to provide the food provenance to the consumer and authorities [13]. As described by Zhong et al. [9], traceability systems in FSCs vary greatly depending on region, government regulations, and digitalization of the FSC. ICT systems as digital twins are able to assist with decision-making, collaboration, scheduling and planning, logistics management, and warehouse management within the FSC [22].

### 2.3. A Definition of Digital Twins

The concept of digital twins first came up during NASA’s Apollo 13 mission in 1970 as the ground team used simulators to provide solutions to the spacecraft crew for landing them safely [23]. The term “digital twin” was first used and defined in 2003 [24]. This concept contains a physical object, a virtual object, and connects data and information from both. NASA formalized the description of digital twins in 2012 and forecasted its potential in the aerospace sector [25]. Here, the digital twin is defined as a multiphysics and multiscale simulation of a vehicle or system, which uses the best physical models, sensor data, and history, resulting in a mirror of the physical counterpart. The discovery that digital twins might be used in a variety of industries other than aerospace accelerated its development [26]; especially, it is an important concept for Industry 4.0 and IIoT.

In this paper, we follow the definition of a digital twin provided by the CIRP Encyclopedia of Production Engineering [27]:

*A digital twin is a digital representation of an active unique product (real device, object, machine, service, or intangible asset) or unique product-service system (a system consisting of a product and a related service) that comprises its selected characteristics, properties, conditions, and behaviors by means of models, information, and data within a single or even across multiple life cycle phases.*

Therefore, a digital twin virtually represents its real-world counterpart, containing all its essential properties [8]. It is based on real-world comprehensive data measurements, which form the digital profile of the physical object or process. Consequently, a digital twin is connected to the real-world object through a continuously updated data flow [28]. Further, the digital twin is able to simulate the relevant processes and kinetics accurately [8]. In this sense, a digital twin may be seen as an ever-evolving digital profile of the past, current, and even future behavior of a process or a physical object and allow to predict uncertainty in the process steps [28].

Defraeye et al. [8] define three common principles to digital twins: Firstly, it must contain all the necessary components and material properties of what it is representing. Secondly, it can reliably and accurately simulate all relevant processes through the product life cycle. Finally, the digital twin should be connected with its real-world counterpart, as this differs a digital twin from simpler models. Communication is preferred to be realized in real-time, but the data could also flow offline.

This is in accordance with Jones et al. [29], who define twinning as the synchronization of the states of the physical and virtual entities. Additionally, the virtual model consists of high fidelity. Bottani et al. [30] expand this, explaining that a digital twin is more than the representation of the physical counterpart since the goal of a digital twin is to replicate all behaviors and relationships of a system and its environment.

**Figure 2:**
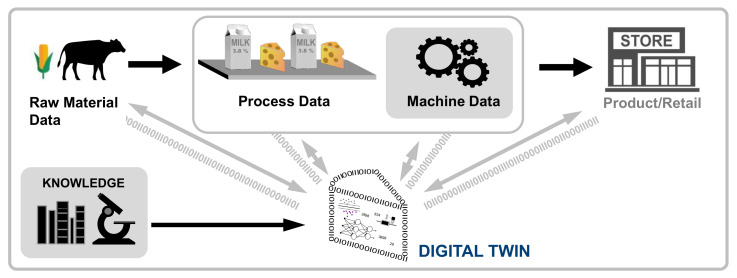
Digital (food) twin basic framework within the context of dairy processing.

Further, five technological components enable digital twins [28]: sensors, integration capabilities, real-world aggregated data, analytical techniques, and actuators. Those technologies are required to aggregate the different available data sources (mainly related to the product and the process) into one comprehensible model of the digital twin as well as support the prediction or analysis with a digital twin. Figure 2 presents the diversity of potential data resources for a digital (food) twin.

A concept closely related to digital twins are CPSs [31,32]. While a digital twin is a digital copy of a product or physical system with the intention of performing real-time optimization, a CPS merges computational and physical processes to seamlessly support humans with intelligence when using machines [33]. CPSs often include digital twins as a base for their decision-making processes [34,35,36].

## 3. Methodology

**Figure 3:**
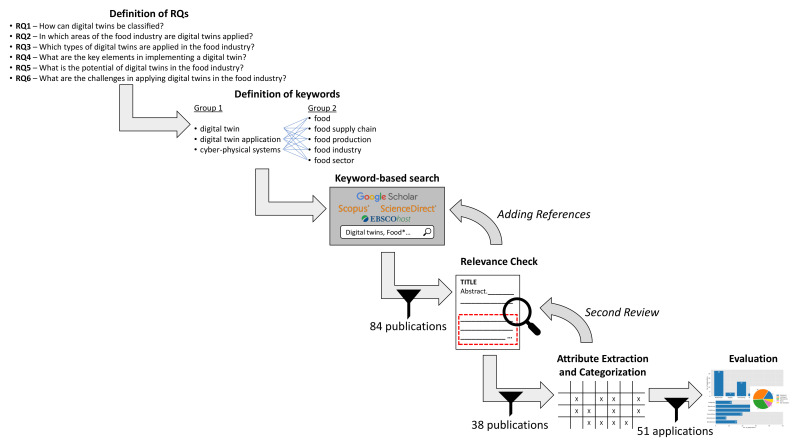
Overview of the methodology for the classification in this survey. Publications found through a keyword-based search were first selected based on the title and abstract. Afterward, the publications were analyzed and relevant publications were categorized in a previously defined taxonomy.

The methodology for the survey integrates methods from the guidelines of Webster and Watson [37] for a structured literature review and Petersen et al. [38] for systematic mapping studies. The research is based on the steps shown in Figure 3. In the beginning, we framed our aim in the form of research questions. We defined exclusion and inclusion criteria and performed keyword-based searches for filtering the articles based on their titles and abstracts. The search method was adapted from [37] to cover a wide range of publications with regards to regions, fields, and publishers. After identifying the set of possible relevant publications, a relevance analysis based on a full paper screening was performed. Subsequently, descriptions and properties of the digital twin applications as well as bibliography data have been extracted and classified as proposed in [38]. In the following, we describe these steps in detail.

### 3.1. Definition of Research Questions

The primary aim of this work is (i) to provide an overview of digital twins applied in the food sector regarding their intended use and (ii) to identify future research areas. According to this goal, we derived the research questions. First, we searched for taxonomies (RQ1) that enable classifying the digital twin concepts that we found according to their application purpose. As we are primarily interested in which area of the food industry (i.e., stages of the FSC) digital twins are applied, we decided to assign the applications found to the stages of the FSC as presented in Figure 1 (RQ2). To better understand the application’s reason for use, we classified the applications found according to our taxonomy (see RQ1) to answer the research question of how digital twin can support the activities in the FSC (RQ3). Further, we analyzed the different types of digital twins that we identified for providing an overview of the different key elements of a digital twin in the FSC (RQ4). Aiming to show the applicability and the benefits of implementing digital twins in different stages of the FSC, we conducted the last research questions. At first, we discuss the potential of digital twins to improve the food industry (RQ5). Then, we discuss the challenges of implementing digital twins in the food sector (RQ6). These considerations lead to the following research questions:

- **RQ1—**How can digital twins be classified?
- **RQ2—**In which areas of the food industry are digital twins applied?
- **RQ3—**Which types of digital twins are applied in the food industry?
- **RQ4—**What are the key elements in implementing a digital twin?
- **RQ5—**What is the potential of digital twins in the food industry?
- **RQ6—**What are the challenges in applying digital twins in the food industry?

### 3.2. Selection Method

To find digital twin applications in the FSC (answering RQ2 and RQ3), we conducted a literature review and included publications available between May and the end of September 2021. Therefore, we searched the databases Google Scholar, Scopus, ScienceDirect, and Academic Search Complete by EBSCO Publishing. We created two groups of keywords: The first group concerned digital twins, including the keywords “digital twin”, “digital twin application”, and “cyber–physical systems”, while the second groups provides the relation to the food sector, i.e., consisted of the keywords “food”, “food supply chain”, “food production”, “food industry”, and “food sector”. The search was performed by combining each of the keywords of both groups.

While we see agricultural plants or farms as part of the FSC (production stage), we did not search directly for “digital twin” AND (“agriculture” OR “agrifood”) since our main focus is on the food quality related to the food processing. Therefore, the food processing and the transportation stages (supply and distribution), as well as the retail, are from special interest rather than the primary food production since the food quality parameters are ultimately adjusted during the processing. After the processing stage, all actions, e.g., cooling, serve to maintain and guarantee the food quality until consumption. Still, we did not discard works related to the agriculture sector when found with our set of keywords.

Additionally, we added publications to our list, which we did not find directly were referred by other publications and possibly relevant for this research (backward search). In the literature search process, we also identified reviews, e.g., [29,39,40,41]. However, as we wanted to avoid the misinterpretation or incorrect reproduction of information, we rather included the original publications or sources of such reviews. Additionally, this ensures that we do not include different points of view for the same application.

Furthermore, we performed a free web search with Google and DuckDuckGo to find examples for digital twins related to the FSC applied in the industry. While this search provided many results, we only included a few of them [42,43,44,45,46,47] as the found information was often not precise enough to analyze in detail required for a classification with our taxonomy.

### 3.3. Analysis Method

The authors selected the publications based on the title and abstract. Additionally, the entire paper was searched to overcome the disadvantages of a keyword-based search. Each publication was reviewed and applications found were classified according to the taxonomy by one of the authors by screening the complete paper. Afterward, each publication, as well as the classification, was reviewed by another of the authors. If an application was classified differently, a third author also reviewed the classification, and the classification was discussed by all authors.

The focus of this work is on digital twin applications related to foods, food products, and their quality. Therefore, the publications needed to contain a specified description of a digital twin application and terms related to “food” (see Section 3.2). Further, we included publications with regards to food products or their quality, meaning we included digital twins of field monitoring applications, animal monitoring applications, and processing machines as well, which we found through the search. We investigated applications that were already realized and implemented as well as concepts for digital twins if the provided description was sufficient enough for the analysis.

A few publications found were located in the periphery of foods, food products, and their quality. For instance, Linz et al. [48] and Tsolakis et al. [49] describe digital twin applications of agricultural machines and robots, whereas the digital twins are used for route planning. Furthermore, Jo et al. [50,51] propose a digital twin for a pigsty to control the energy demand while adjusting the ventilation and temperature. Other publications provided too little information about the digital twin, although they were strongly related to our research, e.g., [31,52]. Since we were not able to classify them, we did not include those in our evaluation.

For some works, we found subsequent publications extending the originally presented digital twin. We added such follow-up publications as dedicated digital twin applications as they develop within the projects or the available information concerning the applications differed in the papers. Further, the originally published digital twin might be sufficient for some applications. In particular, those publications were from Skobelev et al. [36,53,54] concerning (wheat) plants; from Defraeye et al. [8,55], Shoji et al. [14], and Tagliavini et al. [56] regarding fruits; and from Bottani et al. [30] and Vignali and Bottani [57] relating to a pasteurizer.

### 3.4. Selected Studies

**Figure 4:**
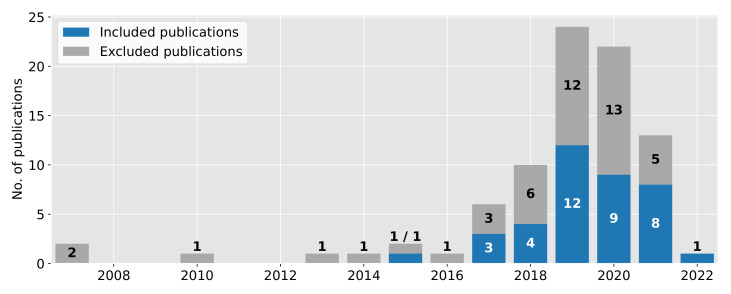
Overview of included (in blue) and excluded (in gray) publications per year. In total, we included 38 of 84 identified publications regarding the analysis of digital twin applications in the food industry.

In total, we studied 84 publications, from which we included 38 publications after the application of the inclusion and exclusion criteria. The publication range spanned works from 2007 to 2022. Worth mentioning is that the publication from Shoji et al. [14] is assigned to 2022 since this is an online first available publication. Figure 4 reveals that the number of publications increased during the last years. In 2019, we observed a peak with 24 publications (12 included). In the years 2020 and 2021, the number of publications is slightly decreasing, counting 22 (9 included) and 13 publications (8 included), respectively. A reason for this decrease could be the COVID-19 pandemic and the inclusion of publications available until the end of September 2021.

**Figure 5:**
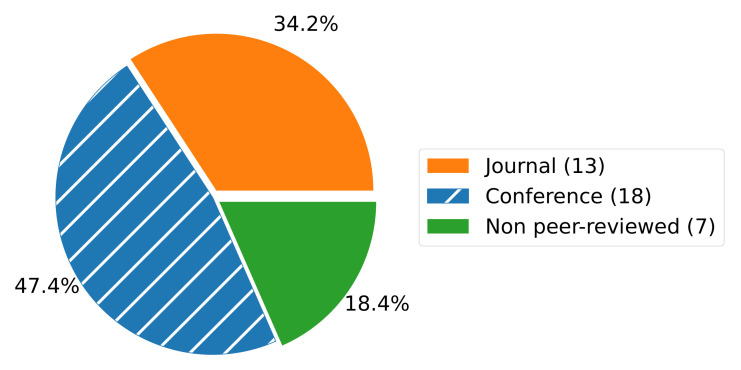
Share of the publication type of 38 included publications.

From the selected publications, the major proportion was originally published at conferences and journals, 47.4% and 34.2%, respectively (see Figure 5). Further, we included non peer-reviewed publications (18.4%) from press releases (2 publications), books, white papers, websites, reports, and project announcements (all one publication each). The inclusion of non-scientific publication types is appropriate for several reasons: Digital twins are still a rather young research topic, particularly in the food sector. In addition, the research is highly driven by the industry since the implementation of digital twins is strongly practice-oriented. However, non-scientific publications often do not provide sufficient details for a classification; hence, this number of included works is limited.

## 4. Results

This section answers the research questions on how to classify digital twins (RQ1), in which areas of the food industry digital twins can be found (RQ2), what types of digital twins are applied (RQ3), and which key elements are required to implement digital twins (RQ4). First, we examined different classification schemes and derived the best fitting taxonomy for our research by combining different existing classification schemes (Section 4.1). Second, we analyze in which activities of the FSC digital twins are applied (Section 4.2). In Section 4.3, we investigate which types of digital twins are applied in the FSC based on our results of RQ1. The classification of all applications included in this section can be found in the Appendix A (see Table A1). Finally, Section 4.4 summarizes the key elements for the implementation of digital twins.

### 4.1. Classification of Digital Twins

Since digital twins have no unique and standardized taxonomy, this section provides an overview of different classification approaches and classifies their relevance for our work. This answers the first research question:

**RQ1—**How can digital twins be classified?

The classification approaches differ in the authors’ focus on digital twins. The authors of [58] differentiate the terms digital model, digital shadow, and digital twin based on the data flow between the physical and digital object. A digital model is defined by a manual data flow between both objects, where the data flows automatically from the physical to the digital object and manually from the digital to the physical object in a digital shadow. The data flow in a digital twin is automated between both objects, which may serve as the controller of the physical object.

In [59], digital twins are classified depending on the application level. The so-called unit-level describes the lowest layer and contains single units of the processing procedure, e.g., equipment or a machine. The system-level consists of several unit-level digital twins and can be understood as a production unit (e.g., a production line), while the System-of-System-level is the highest layer and able to capture complex systems (e.g., the shop-floor management system).

The authors of [39] differ between service categories, meaning the use case of a digital twin. These categories are real-time monitoring, energy consumption analysis, system failure analysis and prediction, optimization/update, behavior analysis/user operation guide, technology integration, and virtual maintenance. They further distinguish the technology readiness level (TRL) between the levels concept, prototype, and deployed. Jones et al. [29] classify digital twins according the product’s life-cycle phases imagination, definition, realization, support/usage, and retirement/disposal.

However, we use a combination of the following two schemes as taxonomy since we are interested in the techniques behind the digital twins and the intended use of the digital twins. According to [8], a digital twin can be statistical, data-driven (intelligent), or physics-based (mechanistic). The first type is based on statistics, where an analytical model is solved with an ordinary differential equation or a simpler analytical equation. The intelligent digital twin is a data-driven model that relies on artificial intelligence (AI) techniques, e.g., machine learning (ML), for model development, calibration, verification, and validation. Mechanistic digital twins are based on physics. Hence, they are also called physics-based digital twins. These models concern all relevant physical, biochemical, microbiological, and physiological processes using multiphysics modeling and simulation. Several authors [4,60] mention that only a mechanistic digital twin is able to mimic the behavior of the real-world counterpart realistically and comprehensively. Therefore, a mechanistic digital twin is preferable for predictions. Worth mentioning is that intelligent digital twins also consider statistical methods. Further, the model parameters used in mechanistic digital twins can be quantified, verified, and validated with statistical and ML methods.

In [8], the authors presented the types in a triangular structure containing the types statistical, intelligent, and mechanistic twins as corners. Therefore, the type of a digital twin could be assigned to corners as well as edges or in between. However, we decided to classify the digital twin applications according to their prevailing type, i.e., there are not any mixed types.

The second classification scheme is similar to [29] since it represents the product’s life-cycle phases. Following the approach by Verdouw et al. [61], digital twins can be used to characterize and simulate the states and behavior of their real-life twins, which do not exist at a specific point in time. Further, digital twins may be used to monitor the current state of items, prescribe desired states, forecast future states, and automatically react to conditions of their real-world counterparts and, therefore, control systems without human interaction. Finally, digital twins are also able to outlast real-world objects, and they can be used to recollect their historical conditions. Worth to mention is that these categories can coexist within the same digital twin application. Table 1 provides a detailed description of the different categories, we used to classify the digital twin applications within the context of this work.

It is notable that the definition of a digital model [58] corresponds to the definition of an imaginary digital twin [61]. Additionally, the categories by [39,61] are similar, but since Pylianidis et al. [39] focus more technical approaches, the approach by Verdouw et al. [61] is used in this work.

### 4.2. Applications of Digital Twins in the Food Supply Chain

In Section 3.4, we observed that the number of publications increased in recent years. Accordingly, the number of digital twin applications increases as well. This section answers the second research question regarding the stage in the FSC where the digital twins are applied:

**RQ2—**In which areas of the food industry are digital twins applied?

**Figure 6:**
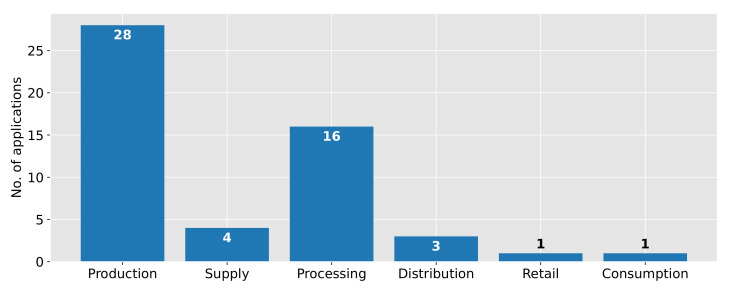
Results of the literature review–Absolute frequency of digital twin applications assigned to stages in the food supply chain. As there are applications [47,56] assigned to several stages, the total number of counts is 53 although 51 applications were found in 38 publications.

Figure 6 provides an overview of the absolute frequency of applications per stage in the FSC. The major proportion of digital twin applications could be found in the production stage, often referred to as agricultural applications (54.90%). Many applications focus on the growth of plants [36,42,53,54,61,62,63,64] or monitoring the condition of animals [23,61,64,65,66]. Further, entire production systems as greenhouses or fields are twinned [34,43,44,61,62,67,68,69,70,71]. Several applications could be described as supportive, e.g., to monitor and control pests [35,65].

The second most frequently assigned stage is the processing stage (31.37%). In this stage, the digital twins mainly concern processing machines, as pasteurizer [30,57] or packaging machines [45,72], or entire processing systems [6,45,73,74,75,76]. A few use cases focus on the optimal product composition or quality [45,46,77].

Applications during transportation, in particular, the stages supply and distribution (7.84% and 5.88%, respectively), determine the quality of fruits and vegetables with a focus on measuring the temperature [8,14,47,55,56,78]. Only one application could be assigned to the retail stage (1.96%), where it is used to determine the quality of fruits and vegetables as well as the remaining shelf-life [47]. Furthermore, one application is assigned to the consumption stage (1.96%). This application aims to twin a consumer to design food products, which are personalized to adapt foods in case of genetic disorders, such as diabetes mellitus [79].

It should be noted that two applications were assigned to multiple stages: While the digital twin of a mango fruit to determine the quality during transportation was assigned to supply and distribution stages [56], the digital twin concept for the determination of the quality of fruits and vegetables was assigned to the distribution and retail stages [47].

### 4.3. Types and Categories of Digital Twins in the Food Supply Chain

In addition to the stages in the FSC, where a digital twin is applied, the applications’ intentions of use are of special interest. In Section 4.1, we specified a taxonomy regarding both the digital twin techniques and the intended use. It is necessary to note that in the case of the taxonomy regarding the intended use, the applications could be classified into several categories. Regarding the digital twin technique, applications could only be assigned to one type. In contrast to the previous Section 4.2, applications were not counted twice if they were assigned to multiple stages of the FSC. Hence, this section answers the third research question:

**RQ3—**Which types of digital twins are applied in the food industry?

**Figure 7:**
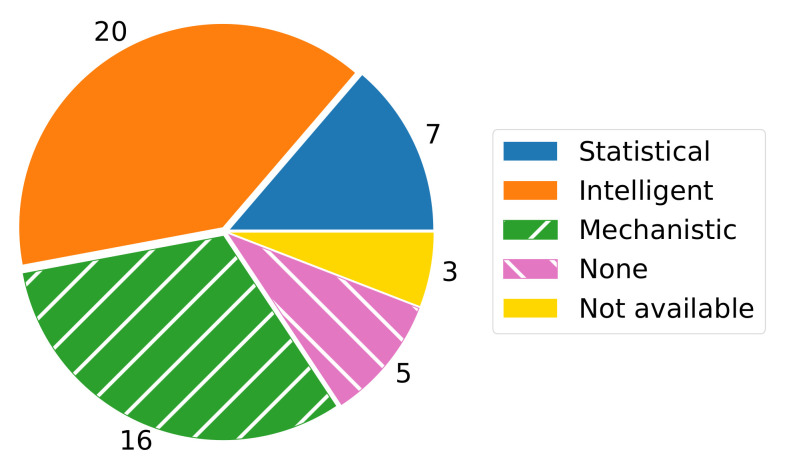
Results of the literature review–Share and absolute frequency of digital twin types found in 38 publications.

Figure 7 shows the classification of the digital twin applications regarding their different types. Most of the digital twin applications are classified as intelligent or data-driven (39.22%). These applications are used for monitoring and controlling plant growth environments, in particular greenhouses or fields [34,42,68,71]; the twinning of plants during growing itself [35,65]; the detection of pests and actions to tackle them [65]; the monitoring of animals [23]; or the determination of shocks and the adaptation of process parameters during potato harvesting [61,78,80]. In addition, applications concern the monitoring of cattle with regards to their health, dairy productivity, or growth (weight gain for meat production) [61,65,66] and the control of food processing parameters [75]. The applications use clustering methods to determine the states and conditions of animals and plants and to classify pests, and further ML techniques to improve the system continuously.

Almost the same proportion of applications are used for simulation, based on mechanistic or physics-based models (31.37%). Many use cases regard either the plant and animal growth in the production stage [36,53,61,62,64,69] or the monitoring of food processing, e.g., a pasteurizer, an ice cream machine, pudding production, malting, or the packaging design concerning special product properties [30,45,57,73,74,76]. More digital twins focus on fruit and vegetable quality during supply by measuring the surface temperature and calculating the pulp temperature based on that [14,56]. All the applications mentioned in this category could be described well with known models.

Further, some applications are based on statistics (13.73%). In this category, many use cases focus on the control of food processing [6,45,46,72]. Other applications regard the design and personalization of food [77,79], or the twinning of a wheat plant [54]. All digital twins are based on statistical methods using means and standard deviations for conclusions and predictions.

It should be noted that there are some applications (15.69% in total), which are not classified to any type [45,65,67] or the classification was not possible due to a lack of information [43,44,45].

**Figure 8:**
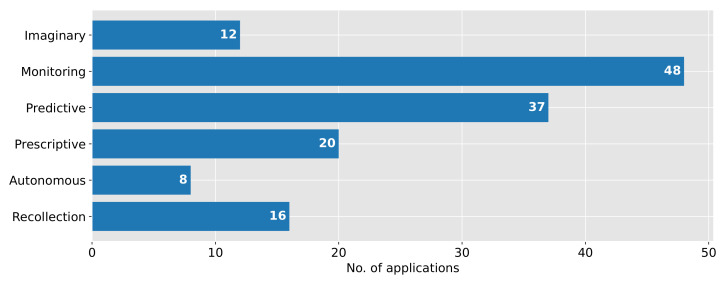
Results of the literature review—Absolute frequency of digital twin categories found in 38 publications. It should be noted that the total number of counts is not equal to the number of applications since they are not restricted to one category.

Figure 8 shows the categorization results of the digital twin applications with regards to their intended use. We observed that nearly all applications (94.12%) are used for monitoring their real-life counterparts. Only three use cases have not been classified in this category; those target applications for the design of new food products and food packaging [45] and the weight gain of cattle for the meat and livestock value chain [66]. We conclude that this observation makes sense since monitoring the physical objects is often the base for further predictions or decision-making. However, only 32 applications (62.75%) are working with real-time data.

Additionally, many applications are used for predictions (72.55%). Use cases, which are not predicting, are mainly used for real-time monitoring and decision-making. These cases concern the detection of pests, the control of plant growth environments based on current growing conditions, e.g., the temperature or humidity [34,44,63,67,68], the monitoring of animals [23,65], the control of food processing [74], and the design of products [45].

The predictions could be used to suggest corrective or preventive actions (39.22%). Since most of the applications found are assigned to the production stage, many prescriptive digital twin applications belong to applications only able to assist in agricultural plants to enhance the quality during growth and harvest processes [42,43,61,62,64,69,70,78,80]. Another prescriptive digital twin is applied in a pudding production system to assist in production planning [73]. Further use cases only recommend actions rather than fully automatizing the system [75]. Examples are the personalized design of foods regarding genetically caused diseases [79] or the design of food packaging [45].

A minor amount of digital twins (15.96%) are integrated into systems working autonomously. The applications automatically control greenhouses by adjusting parameters like temperature or light [34,42,44] or processing plants by controlling, among others, the workflow or specified processing parameters as temperature [45,75].

Some digital twins found were used for forecasting and simulating objects that were presently non-existent (23.53%). This category includes applications for the design of food products and raw materials [53,77] as well as food packaging and production plants [45,67]; applications to predict shelf-life and the food quality [47,56,66]; and applications to control the process flow [6,72,74,75]. The application of imaginary digital twins enables the avoidance of expensive mistakes [75] and detailed planning [67].

Recollective digital twins, that maintain the complete history of physical objects (even if those do not longer exist), can be found in all stages of the FSC (31.37%). Some applications use the stored information for learning and improving the system [34,53,54,61,62,63,68,78,80]. Other applications were implemented to better document the processes and quality parameters of the physical objects [6,30,47,57,74,76,79]. It should be noted that due to a lack of information, many applications could not be classified in this category [23,42,43,44,45,46,56,65,66,67,69,70,71,73,75].

### 4.4. Key Elements for Digital Twin Implementation

In the previous sections, we describe our observations that the implementation of digital twins varies in the different stages of the FSC as well as the intention of use within a specified stage. The major proportion of digital twins are applied in the (primary) production and the processing stage. Especially in the distribution, retail, and consumption stages only a few applications have been found. In addition, different types of digital twins have been found. To investigate how to improve the food quality in the FSC using digital twins, necessary components to apply digital twins need to be identified. Hence, this section answers the following research question:

**RQ4—**What are the key elements in implementing a digital twin?

First of all, there must be a motivation to implement a digital twin. Some digital twins are motivated by production and market reasons, e.g., to cope with a higher demand for more flexibility in the production to adapt to new market demands, such as clients requesting more products that meet unique nutritional standards and packaging sizes [6]. Moreover, the constant increase in business competition challenges companies to look beyond cost reductions and improve quality and productivity [81]. In particular, food processing industries are battling with low-profit margins while being challenged to reduce time-to-market and develop new, flexible processes for a wide range of goods [6]. Another motivation arises out of the demand for more transparency to stakeholders, trust, and ownership of the processes [4]. Finally, some drivers are employee-related, such as offering training based on virtual reality applications that benefit from the data of the models in digital twins [81] and improving employee safety by detecting potential workplace hazards with digital twins [30,81].

Second, the underlying technical infrastructure must be able to support a digital twin [82]. The definition of such a digital model is only possible if a sufficient amount of data is available. Even taking into consideration that a digital twin is able to generate some data of the physical entities (products and processes) through ML, AI, or simulation, still the collection of data from the process through sensors is a critical requirement. Another requirement is related to the communication infrastructure: If real-time analysis is demanded, the infrastructure must support fast exchange of data [23]. Real-time analysis requires a sufficient processing capacity; hence, if real-time analysis is targeted in a project with digital twins, this processing capacity must be available [4]. Thus, a detailed analysis of the available data sources, communication infrastructure, and processing capacity when starting the implementation of a digital twin is required to determine how a digital twin can be enabled.

Third, a digital twin for food production has additional specific requirements compared to digital twins of the production of material goods. Due to the variability of raw materials, these cannot be based only on the processing steps, but must also take into account the chemical, physical or (micro)biological properties of the food [8]. Consequently, either (i) sensors must be available to capture those changes or (ii) simulations based on scientific models of food aging must support information about those intermediate state of the food. Accordingly, the typical, retrospective analysis of machine and process data must be combined with short-term (detection of potential problems), and medium-term data analysis approaches (simulation of product changes) to improve decision making in the food production and tracking of the current state of production at any time with digital twins.

Every digital twin implementation starts with a process design in which all processes and interaction points are mapped that a digital twin will be modeling [28]. Improvements with regard to cost, time, or asset efficiency are augmented in this design process.

**Figure 9:**
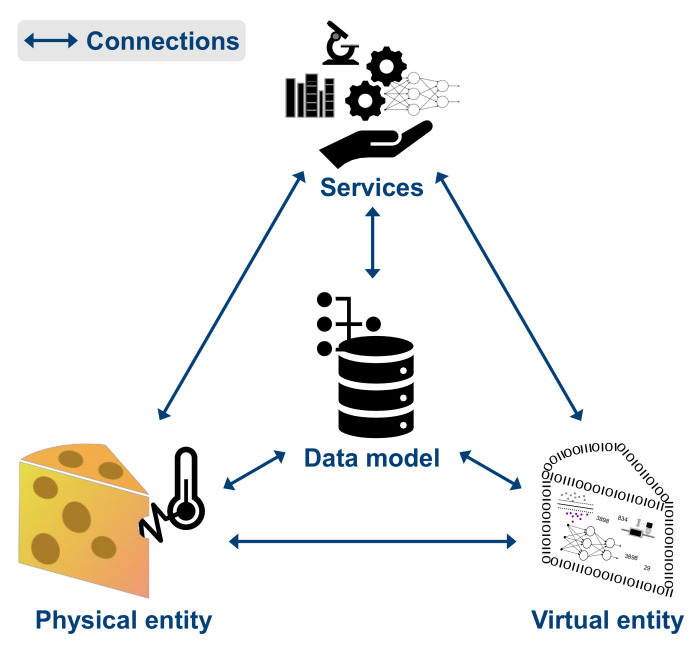
The five-dimensional digital twin concept (adapted from [85]): Digital twins consist of a physical as well as a virtual entity, which are supported by several services. Data are fused and stored centralized. These four dimensions must be connected with each other, creating the fifth dimension.

However, up to now, there is no consensus regarding a generic method in the realization of digital twins that can describe its implementation and the data acquisition from the physical to the virtual object [26]. Therefore, the authors of [83] proposed a digital twin model based on five dimensions (see Figure 9):

- **Physical entity:** The physical world is the basis. The physical entity can be a device or product, a system, a process, or even an organization [84]. It carries out actions following physical regulations and deals with environmental uncertainty.
- **Virtual entity:** The digital model is generated to replicate the physical geometries, properties, behaviors, and rules of the physical entity. Therefore, multiple models can be considered [85].
- **Service platform:** Decision-support analyses support the monitoring and optimization of the physical entity with simulations, verification, diagnosis, and prognosis as well as prognostic [81,84]. Further, the virtual entity must be served with data, knowledge, and algorithms, and the platform itself needs to be served, e.g., with customized software development and model building.
- **Data model:** The data is stored in the data model [81]. Since the digital twin considers multi-temporal scale, multi-dimension, multi-source, and heterogeneous data [84], the data model includes and merges data from the physical entity, the virtual entity, services, and knowledge [85].
- **Information connections:** All dimensions need to be connected to ensure communication and update the information immediately [81]. This enables advanced simulation, operation, and analysis [84].

Barni et al. [86] describe four best practices for the implementation of a digital twin: First, the entire product value chain should be included to ensure data exchange and consistency. Second, the virtual models should be kept dynamic through the development of well-documented methods for model generation and modification. Third, it should be ensured that data from several sources are included to measure the different variables and all essential properties of the physical product and the system (process, actuators, inputs, outputs, and environment) [4]. The exact combination of relevant data is often unknown a priori when the first model is developed; accordingly, the design of the digital twin must offer modularity and scalability [86]. Fourth, long access life cycles should be ensured in order to address a long-term convergence within the physical and the virtual world. The approach by [73] reinforces this through a knowledge acquiring digital twin.

Thus, the accessibility and continuous flow of near real-time data are important [23]. The data generation can be achieved with sensors [8] and the use of IIoT technology [87]. In addition, data processing and data evaluation or interpretation are of high relevance [23], leading to the requirement of sufficient computational performance to handle big data volumes [4]. Therefore, data transfer technologies are required to provide high-speed data gathering from huge amounts of remotely sensor data and transfer it in real-time within a network, e.g., Bluetooth, LoRaWAN or 5G [8].

The core of a digital twin is based on modeling [87]. Therefore, physics modeling (geometrical, mechanical, material, hydrodynamic, and discrete event models), semantic modeling (ML models, deep learning, data mining expert system, and ontology modeling), and model integration (flexible modeling, standard interface, black-box, gray-box, and multiphysics modeling) are used. ML techniques or AI support data analysis and data fusion enabling efficient processing and interpretation of a large amount of data [81,88]; further, those techniques can continuously improve the performance of the system [23]. A key element is a human-machine interface, where users can easily interact with and understand the digital twin’s information [23]. This is particularly important if the digital twin recommends corrective and preventive actions.

In conclusion, the implementation of digital twins requires multidisciplinary knowledge [31], especially from food science. For instance, this includes microbiological, physical, chemical, and engineering disciplines as well as knowledge for efficient process management. Further, ICT is required. Commonly, ICT today is used in the FSC to connect the different stakeholders in the different stages through data exchange. In the future, the support of automated data collection with IoT technology and efficient data analysis, mainly using ML, will have increased importance.

## 5. Discussion

The survey results revealed large differences in the use of digital twins depending on the stages of the FSC: The major proportion of digital twins are applied in production and processing. Further, nearly all applications are used for monitoring, and many applications predict future states of their physical objects. However, only a few digital twins recommend actions or control systems fully autonomously, i.e., refer to prescriptive or autonomous digital twins, respectively. In addition, key elements to implement digital twins were investigated. To better understand the reasons, we primarily discuss the potentials of digital twins in the food industry (Section 5.1). Subsequently, this section discusses the challenges in implementing digital twins (Section 5.2). Section 5.3 closes this discussion with threats to validity.

### 5.1. Potentials of Digital Twins in the Food Industry

As shown in the previous sections, we identified in our literature review several potential ways to optimize the FSC with digital twins. This resulted in the following research question:

**RQ5—**What is the potential of digital twins in the food industry?

In general, digital twins enable data accessibility and advanced analytics in real-time to assist in more informed, efficient, and faster decision-making [89]. Sensor data are fed into a digital twin that runs food process models (i) for providing relevant product process information and operation outputs in real-time for process control, troubleshooting, and supply chain management, as well as (ii) to optimize processes for uniformity, performance, and sustainability or to develop new designs [4,90]. Furthermore, this results in better risk assessment and mitigation strategies based on what-if analyses and simulations [89].

**Improved Use of Sensor Data:** Current approaches in Industry 4.0 focus on the intelligent collection of data with IoT technology and its analysis with ML algorithms [91]. This includes a variety of data sources, including raw material data, machine data, or customer data. Digital twins enable deeper insights due to the use of multi-sensor networks (sensor fusion), where different sensors measure several parameters from different locations [52,60]. As stated before, sensors are required to provide data (environmental, process, machine, etc.) for the digital twins (see Section 4.4). With the development of smart sensors, monitoring the states during processes gets easier and faster [4]. Further, sensors become cheaper, need less power, and transfer the data wireless, which enables their use in more applications, even in mobile settings.

**Combination with Intelligent Packaging:** Intelligent packaging can directly share the quality and current condition of a food product on the packaging during the distribution, retail, and consumption stages [92]. Intelligent packaging consists of intelligent materials or objects, which are defined by their behavior of monitoring “the condition of packaged food or the environment surrounding the food” [93]. Therefore, sensors are integrated into the packaging [92] to monitor, e.g., the temperature, the pH value, the humidity, the pressure on the food, or vibrations during transportation [94]. Further, gas sensors can measure the concentration of carbon dioxide (CO_2_) or hydrosulfuric acid (H_2_S) to allow concluding the current condition of the food [92]. An example of how to produce near zero-cost gas sensors is given by Barandun et al. [95]. Biosensors are able to detect pathogens or toxins in bacteria-contaminated foods [96].

**Combination with AI and ML:** Likewise, integrating nuclear magnetic resonance (NMR) and other spectroscopy methods as well as imaging techniques [4] in conjunction with AI and especially ML enables machine or computer vision. Such algorithms can analyze the food and are able to determine its composition, condition, and quality issues as spoilage, contaminants, or defects [97,98]. Furthermore, by placing virtual sensors on the digital twin model, sensor data from locations that would usually not be accessible to sensors can be generated [8]. Virtual sensor data are software-based outputs of fused data from physical sensors [99]. The application of physical sensors is limited by noise, interference, or unfeasibility due to spatial conditions [99] or locations difficult to access [8]. Virtual sensors provide data measurements of parameters or locations, which are physically not measurable [99]. This application enables the detailed prediction of food losses and the remaining shelf-life of the food products [60].

**Optimizing Production Planning:** Further, production planning can be optimized with ML in this context [100]. The industry demands the possibility to adapt to current market demands as unique nutritional standards and packaging sizes and, therefore, require a higher production flexibility [6]. This means not only being able to produce a wide range of products also counting with the capacity to reschedule the production dynamically [81]. The analysis and prediction of SC disruptions can be used to assist this [5,101], although the mentioned references focus on more economic aspects of these disruptions. Proactive adaptation improves system performance as it forecasts adaptation concerns (e.g., through identification of patterns in historical data) and reacts either by preparing an adaptation or adapting [102]. Autonomous systems can respond to changes in the state during ongoing operation, while digital twins can integrate a variety of data like environment data, operational data, and process data [26,103]. This also includes supplying different stakeholders in the FSC with actionable real-time data, such as the remaining shelf-life for each shipment (based on the product’s physical, biochemical, microbiological, or physiological states), on which logistics decisions and marketing strategies can be adjusted [8,89].

**Support for Predictive Maintenance:** Another use case is predictive maintenance of machines [104]. Digital twins are able to show the evolution of the process in each element of a production or processing machine without the need to halt the process or open the system to examine its state physically [30]. Faults in the system can be spotted significantly earlier thanks to intelligent data analysis [89], leading to more efficient approaches for predictive maintenance, which is made before faults or failures occur [105]. This can be considered in production planning and decrease down-times. Further, virtual reality and augmented reality can be based on digital twins and support training and maintenance or repair of machines [90,106].

**Support for Product Development:** Digital twins are also useful during product and process design, where actual monitored sensor data allow to check for conformance of the product specifications with the design intent and customer requirements [8,107,108]. Additionally, tests on prototypes can be replaced by simulations on the digital twin, which results in a reduction of costs, time, and resources [77,105,109]. Regarding the complete product life cycle, digital twins also respect the disposal of the packaging and food remains and, therefore, consider sustainability aspects [105]. Aiming to achieve a sustainable FSC, digital twins can optimize the environmental impact as a consequence of the growth of production systems [110].

**Improving Collaboration:** Digital twins facilitate the collaboration of cross-functional teams [89]. They can be used to clarify specifications with suppliers and optimize designs. If the company develops a new digital twin with every product, each model will comprise data on the precise components and materials used in the product, configuration options specified by end consumers, as well as process conditions experienced during production [111]. Moreover, digital twins are able to assist in terms of personalized nutrition by adjusting product recipes in response to changes in consumer preferences; designing products with a specific chemical composition, nutritional value, and functional orientation; and developing functional, specialized products tailored to the needs of small groups of people that will assist in lowering the risks of disease in those who already have it, as well as satisfy the demands of those who want to tailor their diet to their specific needs [77,79].

**Enhance Food Safety:** Furthermore, digital twins can enhance food safety by improving product traceability [112] through the possibility to identify problems in real-time and to record this by storing shipment condition data [8]. Worth mentioning is the approach by Botta et al. [112], combining a blockchain-based verifier with the digital twin application to validate and secure the data. Another framework combining the blockchain technology with digital twins is proposed in [113]. The aim of this framework is the secured data sharing and exchange during collaboration of several digital twins, e.g., in logistics tasks and risk prediction. Further, digital twins could assist regulatory organizations with providing useful data to avoid delays in import and export or companies during the application of the Hazard Analysis and Critical Control Points (HACCP) concept to suggest control points and remedial actions [8].

### 5.2. Challenges in Implementing Digital Twins in the Food Industry

The implementation of a digital twin consists mainly of the following key elements: a real-life object or process, which should be twinned; a virtual model of the real-life counterpart, including all its essential properties; and a linkage between both [8,30,85]. Further, technical components are required to sense the physical entity and adjust the virtual entity accordingly or to store and process data. The extent of applications differs in the stages of the FSC, although digital twins provide potentials in the food industry as discussed in the previous Section 5.1. Hence, this section addresses the following research question:

**RQ6—**What are the challenges in applying digital twins in the food industry?

One of the major challenges of implementing digital twins is the lack of a general method, which describes how to gather the information from the physical to the virtual object [4,26,90]. Koulouris et al. [6] state that the specific characteristics of the food sector and high-value product industries, such as specialized equipment, component complexity, and high-quality standards, are responsible for the delay in the adoption of process simulation for design and modeling. Thus, the individual projects for implementing a digital twin lead to higher investment costs due to the diversity of approaches and, therefore, are particularly challenging in smaller companies and poorer countries [4,26,114]. In the following, we describe further, specific challenges.

**Complexity of Food:** The complexity and variability of raw materials and their properties used to create food products, and the limited shelf-life not only of food raw materials but also the products made of it are limiting the application [4,6]. Further, plants, processes, and knowledge are continuously changing environments, forcing the related digital twins to improve permanently [73]. Moreover, the lack of “multi-spatial/time scale models” from the current modeling technologies limits the representation of behaviors, features, and rules at the diverse levels and granularities of the spatial scale and the characterization of the dynamic process of physical entities from different time scales [84].

**Absence of Physicochemical Models:** The absence of good physicochemical data is another major impediment to the use of modeling and simulation tools [6]. For instance, food processing faces a wide range of foods with complex properties, hard to calculate or even to predict, such as molecular weight, pH, or water activity, and not so well understood thermodynamics. Furthermore, the kinetics of biological and chemical processes need to be understood and made calculable as physics-based models [4]. This effect is intensified by production mixes, technology variability, and the unpredictability of the physical solution [86], resulting in complex integration of different modeling methods [4]. However, process models can already be incorporated to estimate the energy and material requirements and expected process yield during the food processing [6].

**Complexity:** Depending on the complex integration of different methods in the digital twin application, the maturity of prescriptive analytic techniques might become a risk due to unreliability, thus a barrier to implementing a digital twin [81]. Further, the complexity of the digital transformation in the FSC requires step-by-step implementation, which takes several years until a productive state is achieved. Here, on the one hand, data security and validation need to be considered [31]. On the other hand, realizing autonomous systems need to pay attention to legislation, in particular hygienic requirements as well as traceability of the system’s decision.

**Missing Technological Infrastructure:** Another challenge is that only by advancing sensor, communication, and data processing technologies, real-time interaction between actual and virtual twins can be achieved [86]. The systems themselves have to enable the implementation of digital twins, i.e., their properties must be known or observable, as well as they have to provide high-quality data [82]. In particular, production and processing machines need to be upgradeable, which may lead to higher investment costs [115]. Further, there are studies on remotely food monitoring during distribution, retail, and consumption [116,117]. However, technologies such as radio-frequency identification (RFID) or near-field communication (NFC), which would support the collection and transfer of data [97,98,117,118] are not widely applied for this purpose yet [119].

**Missing Organizational Readiness:** Further, there might be obstacles with regard to the culture in the food industry. Firstly, the human acceptance of novel and advanced technologies challenges the application of digital twins [109], especially as the competencies of the employees in ICT might be heterogeneous. For example, the survey “Nutrition 4.0-Status Quo, Opportunities, and Challenges” by Germany’s digital association Bitkom and the Federation of German Food and Drink Industries (BVE) showed that 88% of the more than 300 surveyed companies in the food industry consider a lack of ICT competencies of their employees as a critical issue [120]. Secondly, the food production and processing industry is partially highly automated; however, in general, the industry is rather conservative with introducing new technology that automatically controls processes [31]. Lastly, the risk of lower attention to the real-world system and the dependency on the recommendations by digital twins need to be considered [52]. This might be a reason for the small amount of prescriptive and autonomous digital twins.

**Missing Knowledge of Employees:** The required expertise of knowledge becomes a real challenge for project teams [114]. In order to address the requirements resulting from the key elements, multidisciplinary knowledge is required [84]. This includes expert, plant, machine, and product knowledge [31]. Additionally, the ICT infrastructure, as well as their establishing and organization, play important roles [31,81].

**Distribution of the FSC:** The size of the system, which should be twinned, is further a challenge [81]. Since FSCs are often distributed across several entities, numerous legal regulations must be considered [31]. Furthermore, the entire environment must be taken into account with respect to the complete implementation of all required connections within the digital twin. These connections (including explicit and invisible ones), internal logic interactions, and external relationships given in the physical world are difficult to be reproduced virtually [84]. Thus, the implementation and improvement of a digital twin is a long process to achieve high effectiveness of the digital twin. However, because the intricacy of the interactions and processes makes it difficult to capture various characteristics of real-world supply chains, their models created are often simplified [121].

### 5.3. Threats to Validity

We used a well-structured approach for the literature review to provide a structured analysis. Each identified paper was read and classified by at least two authors of this work; unclear classifications were discussed by all authors. This significantly helps to reduce human bias in the process. However, some threats to validity still exist, which we discuss in the following.

The choice of keywords might be restricted. While this survey revealed many use cases in the production stage, often referred to as agriculture or agricultural application, we did not explicitly search with keywords concerning digital twins in agriculture. This may lead to a lower outcome of search results and the missing of relevant publications and applications. However, it is common practice to narrow the scope for being able to handle a topic’s complexity, and we clearly describe the used keywords in Section 3.2.

In addition, we used “cyber–physical systems” as a keyword since those systems often integrate digital twins. This search revealed publications, which have not explicitly mentioned the term “digital twins”. As the term itself is still relatively young, some publications might have been describing digital twins in a CPS without using the term. Moreover, it was not always possible to differentiate between simpler digital models/representations and digital twins. As a result, relevant applications may not have been taken into account.

Further, the free web search using a search engine (rather than a scientific database) provided many results, including scientific publications, press releases, offered product ranges, project announcements, explanation videos, and more. Despite the great efforts we have made for this survey, we were not able to analyze all search results in detail and to the fullest extent. Therefore, some applications may have been omitted. However, our analysis also showed that non-scientific publications from industry often missed the required depth of detail to analyze and classify those publications thoroughly; hence, we assume that the additional contribution would be limited.

Each publication was initially analyzed by one of the authors of this work. We followed a well-defined approach. Still, as humans are involved, the presence of subjective bias cannot be entirely excluded. To limit this risk, we double-checked each analysis by at least a second reviewer for each paper. In case of deviations, we discussed those publications with all authors.

In particular, some applications were not possible to classify clearly to the stages of the FSC, defined in Section 2.1. This is caused by different definitions of the FSC and FSC structure or by the unspecified description of the referred stages in other publications. Others might argue that our FSC structure is not appropriate or not flexible enough for this classification, e.g., in the case of fresh fruit SCs. However, this paper aims to provide an overview of digital twin applications in the FSC. Therefore, a clear structure of the FSC is required, and the structure in this paper merged the most frequently used stages.

## 6. Related Work

This work investigates the use of digital twins in the food industry, represented by the FSC, and studies the challenges and potentials of digital twins in the FSC. In this section, we provide an overview of related publications from the area of digital twins.

While the concept of digital twins and their technical capabilities are still in their infancy, literature reviews on digital twins exist. However, some reviews are not focused on foods, the food industry, or at least parts of the FSC but were taken into account in this work. Jones et al. [29] characterized digital twins in general by determining the key terminology of digital twins. Therefore, they examined intentions of use and applied technologies. Finally, the authors identified research gaps to apply digital twins, concluding a review limited to more unified domains would be better. Concerning digital twins in SCs and logistics, Abideen et al. [122] conducted a literature research on ML integrating digital twins. The authors further propose a framework for this. However, their focus is on digital twins used to improve logistics not considering the food sector at all or the food’s quality in detail. The work of Klerkx et al. [109] investigated digitalization in agriculture from a social-science perspective. In that sense, they review several related technologies, e.g., IoT, blockchain, and digital twins, among others, with regards to social aspects as the farmer’s identity and skills; ethics with regards to power supply and consumption and data privacy; and economics.

Other works focus on a specific stage of SCs. Pylianidis et al. [39] surveyed the implementation of digital twin use cases in agriculture in particular and over all disciplines in general. Similar to our work, they classified the applications with regards to the discipline and the service category, according to the stage of the FSC and the digital twin type, respectively. They further considered the TRL, i.e., differentiate concepts, prototypes, and deployed digital twins. Additionally, Verdouw et al. [61] provided a scheme, which is used in our work. However, they focused only on agricultural applications as animal monitoring and crop management, which we included as well. Kritzinger et al. [58] differentiated the integration level concerning the data flow between the physical and virtual entity and concluded that the terms digital model, digital shadow, and digital twin are used interchangeably. The authors further regarded the type according to the TRL. They revealed that digital twins in manufacturing are most often present, but the work did not focus on food processing.

A more all-encompassing view on the agri-food SC is presented in the work of Tebaldi et al. [40], including the SC stages supply, processing, and distribution (according to our taxonomy in Section 2.1). For the sake of completeness, we included the applications mentioned there in our work. Further, the works of Ivanov et al. [101] and Burgos and Ivanov [5] took entire SCs into account concerning the analysis of disruption risks. Therefore, ref. [101] proposed a digital twin framework to analyze risks, to predict resilience, and to optimize the SC in order to avoid critical disruptions. The impact of the COVID-19 pandemic on FSCs is analyzed using a digital twin in [5].

However, to the best of the authors’ knowledge, there is no publication that discusses and reviews the application of digital twins in the whole FSC. Further, the derived research challenges to improve the integration of digital twins into the FSC, which acts as a kind of research agenda for the community, are unique in literature.

## 7. Conclusions

This work investigated the challenges and potentials of applying digital twins in the food industry. Therefore, we conducted a literature review concerning 51 digital twin applications and assigned them to previously defined stages of the FSC. The survey revealed that the major proportion of use cases is implemented in the production, often referred to as agriculture, and processing stages (28 and 16 applications, respectively). In addition, only a few use cases are deployed in the supply, processing, retail, and consumption stage (9 applications in total).

Classification: Further, we classified the applications regarding their underlying model and the intention of use. Most of the digital twins are based on intelligent or mechanistic models (20 and 16 applications, respectively). A minor amount uses statistical models (7 applications). Nearly all of the examined digital twins are used for monitoring the physical counterpart (48 applications). Additionally, 37 applications calculate predictions. However, only a minor amount of digital twins recommend actions or assist in autonomous system control (20 and 8 applications, respectively). Few applications are referred to imaginary digital twins (12 applications). A few more use cases maintain the history (16 applications), but uncertainty due to a lack of information must be considered in this category.

Identified challenges: The main challenges of integrating digital twins within FSCs stem from the difficulty of collecting high-quality physiochemical data and integrating digital twins into existing supply chain structures [6]. High-quality physicochemical data is required for the use of digital twin modeling and simulation tools. However, it is challenging to collect and process this type of data due to food processes having inadequately described properties and difficult to calculate or predict variables, among other factors. Effective data models that can accommodate this variability are required; however, there are currently no commercially data models available that can integrate different modelling methods on different scales [86]. Further, the lack of multidisciplinary knowledge is challenging the application [31]. In order to tackle this, new research perspectives, such as Food Informatics [123], need to be deployed.

Identified future work: In order to assist data accessibility, novel and cheaper sensors are developed, enabling them to be integrated into the food packaging [92]. In conjunction with other related technologies as blockchain, this provides more possibilities to monitor the food’s condition during the later stages [124]. This leads to a transformation of the FSC with digital twins that potentially offer greater transparency, improved traceability, reduced disruption risk, and optimized processing. In addition, digital twins allow to sense and monitor parameters and states at difficult-to-access or even inaccessible locations, e.g., pulp or machines, by providing the ability to place virtual sensors. Finally, through the creation of digital human clones, food production can become more individual and personalized with regards to human health [77,79].

## Figures and Tables

**Table 1:** Digital twin taxonomy (based on [8,61]).

Type	Description	
Statistical	Solving a simple analytical equation or an ordinary differential equation (ODE) for calculations with the generated data.	[8]
Intelligent	Use of AI techniques, e.g., ML, for model development, calibration, verification, and validation.	
Mechanistic	Performance of multiphysics modeling and simulation to capture the relevant physical, biochemical, microbiological, and physiological processes.	
Imaginary	Simulates objects that do not physically exist in the real-world at the given time.	[61]
Monitoring	Monitors the current state and behavior of a real-life, physically existing counterpart.	
Predictive	Projects future states and behavior of a physical object based on real-time data.	
Prescriptive	Are able to intelligently recommend corrective and preventive actions while using the results of monitoring and predictions.	
Autonomous	Control autonomously the behavior of the real-world counterparts without human intervention.	
Recollection	Maintains the complete history of physical objects, which no longer exist in real-life.	

## Data Availability

There is no supplemantary data. Detailed information concerning the classification are provided in the Appendix A.

## Appendix A

The survey in this work was based on a systematic mapping. Therefore, applications were classified according to the taxonomy proposed in Section 4.1. Table A1 provides a complete overview of the applications found in the literature and included in this work. Few publications contained several applications. Therefore the use cases can be distinguished through this table. Further, it reveals the FSC stage, the applications were assigned to, and how we classified the applications in detail.

**Table A1:** Overview of the applications found in the literature and included into this work and their classification according to the taxonomy in Section 4.1. Please note: ‘X’ marks true, ‘n.a.’ that the information was not available. Further, the following abbreviations are used in the table header: Ref.–Reference; rt–real-time; stat–statistical; int–intelligent; mec–mechanistic; ima–imaginary; mon–monitoring; pred–predictive; pres–prescriptive; auto–autonomous; and rec–recollective.

Ref.	Application	Stage	rt	stat	int	mec	ima	mon	pred	pres	auto	rec
[6]	Beer brewery	Processing	X	X			X	X	X			X
[8]	Mango (fruit)	Supply				X		X	X			
[14]	Fruits and vegetables	Distribution				X		X	X			
[23] ${}^{1}$	Animal monitoring	Production	X		X			X	n.a.	n.a.	n.a.	n.a.
[30]	Beverage pasteurizer	Processing	X			X		X	X			X
[34]	Greenhouse	Production	X		X			X		X	X	X
[36]	Wheat plant	Production	X			X		X	X			
[42]	Greenhouse	Production	X		X			X	X	X	X	n.a.
[43]	Greenhouse	Production	n.a.	n.a.	n.a.	n.a.		X	X	X		n.a.
[44]	Greenhouse	Production	X	n.a.	n.a.	n.a.		X			X	n.a.
[45]	AMWAY (Product design)	Processing	X				X				X	n.a.
[45]	KRONES (packaging design)	Processing				X	X		X	X		
[45]	Beverage plant (filling)	Processing	X	X				X		X	X	n.a.
[45]	Cheesery plant	Processing	X	n.a.	n.a.	n.a.		X	n.a.	n.a.	X	n.a.
[46]	Processing plan (chocolate bars)	Processing	X	X				X	X			n.a.
[47]	Fruits and vegetables	Distribution/Retail			X		X	X	X			X
[53]	Plant	Production	X			X	X	X	X			X
[54]	Wheat plant	Production		X				X	X			X
[55]	Mango (fruit)	Supply				X		X	X			
[56]	Mango (fruit)	Supply/Distribution				X	X	X	X			n.a.
[57]	Tube pasteurizer	Processing	X			X		X	X			X
[61] ${}^{2}$	Potato (vegetable)	Production	X		X			X	X	X		
[61] ${}^{3}$	Animal monitoring (cow)	Production			X			X	X			
[61] ${}^{4}$	Greenhouse	Production	X		X			X	X	X		
[61] ${}^{5}$	Organic vegetable farming (grow and harvest lettuce)	Production	X		X			X	X	X		X
[61] ${}^{6}$	Animal monitoring (pig)	Production				X		X	X			
[62]	Hydroponic farm	Production	X			X		X	X	X		X
[63]	Potato plant	Production	X		X			X		X		X
[64]	Aquaponic system	Production	X			X		X	X	X		
[65] ${}^{7}$	Dairy Monitor (cow)	Production	X		X			X	X			n.a.
[65] ${}^{8}$	Open PD (plant desease detection)	Production						X				n.a.
[65] ${}^{9}$	INSYLO (silo’s stock monitoring)	Production	X					X				n.a.
[65]	OliFLY (pest traps for olive fly)	Production	X		X			X				n.a.
[65] ${}^{10}$	BeeZon (apiary monitoring)	Production	X					X				n.a.
[66]	Animal monitoring (cow)	Production			X		X		X			n.a.
[67]	Vertical Farm	Production					X	X				n.a.
[68]	Crop management (irrigation system)	Production	X		X			X		X		X
[69]	Aquaponic system	Production	X			X		X	X	X		n.a.
[70]	Orchard production system	Production	X		X			X	X	X		n.a.
[71]	Crop management	Production	X		X			X	X			n.a.
[72]	Processing plant (water filling)	Processing		X			X	X	X			
[73]	Processing plant (pudding)	Processing	X			X		X	X	X		n.a.
[74]	Malthouse	Processing				X	X	X		X		X
[75]	Processing plant (Ketchup)	Processing	X		X			X	X		X	n.a.
[75]	Processing plant (milk powder production)	Processing	n.a.		X		n.a.	X	X	n.a.	n.a.	n.a.
[75]	Processing plant (cheese)	Processing	X		X		X	X	X		X	n.a.
[76]	Ice cream machine	Processing				X		X	X			X
[77]	Meat product	Processing		X			X	X	X			
[78]	Potato (vegetable)	Supply	X		X			X	X	X		X
[79]	Consumer	Consumption		X			n.a.	X	X	X		X
[80]	Potato (vegetable)	Production	X		X			X	X	X		X

^1^https://www.cainthus.com/ accessed on 23 December 2021; ^2^ https://www.iof2020.eu/use-case-catalogue/arable/within-field-management-zoning accessed on 23 December 2021; ^3^ https://www.iof2020.eu/use-case-catalogue/dairy/happy-cow accessed on 23 December 2021; ^4^ https://www.iof2020.eu/use-case-catalogue/vegetables/chain-integrated-greenhouse-production accessed on 23 December 2021; ^5^ https://www.iof2020.eu/use-casecatalogue/vegetables/added-value-weeding-data accessed on 23 December 2021; ^6^ https://www.iof2020.eu/use-case-catalogue/meat/pig-farmmanagement accessed on 23 December 2021; ^7^ https://www.connecterra.io/ accessed on 23 December 2021; ^8^ http://www.openpd.eu/ accessed on 23 December 2021; ^9^ https://www.insylo.com/ accessed on 23 December 2021; ^10^ https://www.beezon.gr/el/ accessed on 23 December 2021.
